# Effect of Microstructure on Thermophysical Properties of Heat-Treated Duplex Steel

**DOI:** 10.3390/ma14206043

**Published:** 2021-10-13

**Authors:** Piotr Koniorczyk, Judyta Sienkiewicz, Janusz Zmywaczyk, Andrzej Dębski, Mateusz Zieliński, Marek Preiskorn

**Affiliations:** Faculty of Mechatronics, Armament and Aerospace, Military University of Technology, ul. gen. S. Kaliskiego 2, 00-908 Warsaw, Poland; judyta.sienkiewicz@wat.edu.pl (J.S.); janusz.zmywaczyk@wat.edu.pl (J.Z.); andrzej.debski@wat.edu.pl (A.D.); mateusz.zielinski@wat.edu.pl (M.Z.); marek.preiskorn@wat.edu.pl (M.P.)

**Keywords:** duplex stainless steel, heat treatment, microstructures, thermophysical properties, thermal diffusivity, specific heat capacity, thermal expansion

## Abstract

The purpose of this study is to investigate the effect of heat treatments and resulting changes in microstructure on the thermophysical properties of commercial 1.4462 duplex stainless steel. Three types of heat treatment and a raw sample were used. In the first heat treatment, a duplex steel bar was annealed in an air atmosphere furnace for one hour at 1200 °C and then quickly cooled in water (1200 °C + water). The second heat treatment was the same as the first, but afterwards, the bar was annealed in an air atmosphere furnace for 4 h at 800 °C and then slowly cooled down in the furnace to room temperature (1200 °C + water + 800 °C). In the third heat treatment, the duplex steel bar was annealed in the furnace in an air atmosphere for one hour at 900 °C and then slowly cooled in the furnace to room temperature (900 °C). As a result, the weight percentages of ferrite and austenite in the samples achieved the following ratios: 75:25, 65:35 and 44:56. Light microscope examinations (LM), scanning electron microscopy (SEM), Vickers micro-hardness measurements and thermophysical studies using a laser flash apparatus (LFA), differential scanning calorimetry (DSC) and push-rod dilatometry (DIL) were performed to reveal the microstructure and changes in thermophysical properties including thermal diffusivity, thermal conductivity, thermal expansion and specific heat. Along with presenting these data, the paper, in brief, presents the applied investigation procedures.

## 1. Introduction

Duplex stainless steels (DSSs) are widely used in the offshore oil, gas and petrochemical industries to build chemical tankers, desalination plants, chemical and petrochemical processes lines, pipelines, and oil and gas separators. These steels have an advantage over conventional austenitic stainless steels due to their high resistance to chloride-induced stress corrosion cracking. They are also resistant to pitting and crevice corrosion and are approximately twice as strong as common austenitic steels. The term “duplex steel” denotes a steel grade whose annealed structure consists of approximately equal parts of austenite and ferrite. Duplex steels contain 22 to 27% chromium, 3 to 7% nickel and up to 4.5% molybdenum [1]. Such good physical and mechanical properties of duplex stainless steels occur only in the temperature range from −50 °C to 300 °C. To summarize, DSSs solidify as “δ” ferrite and then transform partially through a solid-state reaction at temperatures between 1200–1400 °C into austenite at the grain boundaries. It is well known that DSSs’ strength is not significantly affected by the ferrite/austenite phase ratio but rather mainly by the presence of quite brittle intermetallic phases [2]. Owing to the high alloy content in the DSSs, their precipitation behavior is greatly complex [2,3,4,5,6,7,8,9,10]. It is also worth noting that those phases occur only in the ferritic phase, as the diffusion rates for alloying additives in ferrite are approximately 100 times higher and their solubilities 100 times lower than those in austenite [3]. Taking into account kinetic formation, precipitation processes can be controlled by the element solubility and diffusion in DSSs. Phases that are dependent on element solubilities, such as austenite, carbides and nitrides, cannot be eluted through technically practicable cooling processes; the χ- and σ-phases that their formation depends on the diffusion of may be released by rapid cooling. Generally, the precipitation of a large variety of secondary phases in DSSs occurs in the temperature range of 300–1000 °C. This range can be divided into two separate temperature regions: (a) below 600 °C, where nitrides, carbides, and χ– and σ-phases develop; (b) from 600–1000 °C where “475 °C embrittlement” associated with the decomposition of δ ferrite into Cr-rich α’- and Fe-rich α-phases is observed [3].

The effects of intermetallic precipitation on the mechanical properties of DSSs have been studied intensively by various authors over the last two decades [2,7,11]. Topolska et al. [7] studied the effects of microstructure on the impact toughness of duplex and super duplex stainless steels. They concluded that the amount of ferrite is higher after aging at 900 °C than at 800 °C due to the direct transformation of ferrite into the σ phase at higher temperatures. Performed tests have shown the negative effect of precipitates, mainly σ phase, on the plasticity of 1.4462 DSS. Toughness of DSS decreased considerably when some amount of σ phase appeared in the steel microstructure. 

While the influence of the microstructure on the mechanical properties of DSS in delivery condition is relatively well known and described in the literature, the thermophysical properties are still the subject of research. Lecomte-Beckers et al. [12] investigated the thermal characteristics of thermal diffusivity, thermal conductivity, specific heat and thermal expansion in DSS Uranus 76N non-rolled slabs in longitudinal and transversal directions. The temperature characteristics of the specific heat revealed reverse austenitic transformations appearing at 516 °C and at 1000 °C. Klancnik et al. [13] compared DSC experimental data for SAF 2205 DSS and AISI 304 LN austenitic steel with the results of thermodynamic calculations using the CALPHAD (CALculation of PHAse Diagrams) method. Regarding the calculated heat capacity diagrams for both steels, relatively good agreement was obtained between the thermodynamic calculation and experimental results. Riad Harwill et al. [14] investigated eleven different DSSs to study the effect of microstructure on the thermal diffusivity and heat capacity values. Results showed that the ferrite content in DSS increased with temperature at equilibrium state and there was no significant effect of ferrite content in DSS on the thermal diffusivity value up to 500 °C.

As DSSs find newer and newer applications, e.g., in the arms industry for the production of barrels for small arms, new methods of heat treatment are being proposed. These studies concern samples of 1.4462 DSS subjected to heat treatment in Polish armaments plants. The aim of the present study is to reveal the effect of thermal cycles on structural changes and mechanical and thermophysical properties of 1.4462 DSS. Specifically, it focuses on studying the influence of heat treatment and the resulting changes in microstructure on the mechanical (mainly hardness) and thermophysical properties of the commercial 1.4462 DSS.

## 2. Materials and Methods

### 2.1. Materials

The as-delivered material was in the form of 1.4462 DSS bars with a diameter of 20 mm and a length of 200 mm. The nominal composition (wt.%) of the 1.4462 (X2CrNiMoN22-5-3) DSS alloy is shown in Table 1. The DSS is a two-phase steel with an austenitic–ferritic structure. During heat treatments, DSS is prone to microstructure changes and precipitation of intermetallic phases. These precipitates are very harmful for the mechanical properties and corrosion resistance of the steel. The structure of the 1.4462 DSS as received was obtained by rolling, and formed a fine elongated lamellar structure of austenite and ferrite. In the literature, the ferrite content in 1.4462 DSS is estimated at 44–47% [7]. 

### 2.2. The 1.4462 DSS Phase Diagram

The 1.4462 DSS alloy is one of a group of DSS steels in which many different solid-state reactions can take place at temperatures higher than 300 °C. These reactions lead to the formation of various precipitates and dissolution processes, which may cause changes in the thermophysical properties of the material. Undoubtedly, some of those precipitations have a negative effect on the mechanical properties of the resulting steel, i.e., strength and hardness or corrosion resistance [7]. 

When DSS reaches temperatures above 300 °C, one of the first effects is the so-called “475 °C embrittlement”. In the temperature range of 500–900 °C, DSS undergoes changes in microstructure and the precipitation of intermetallic phases occurs. The short precipitation time of these deposits should be taken into account when planning the DSS heat treatment [7].

**Figure 1:**
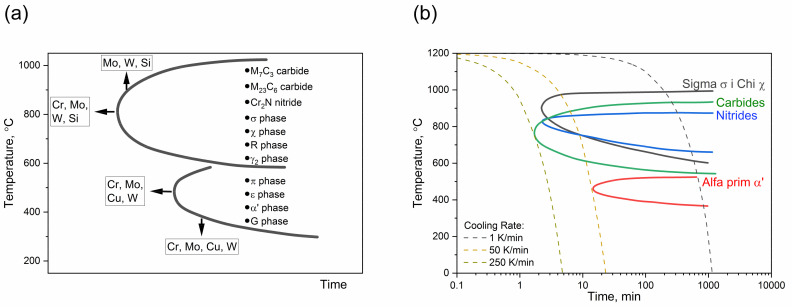
(**a**) Schematic TTP diagram for secondary phases in DSSs; (**b**) Isothermal Precipitation Diagram for DSSs, annealed at 1050 °C [16].

A schematic time-temperature-precipitation (TTP) diagram is shown in Figure 1a. This diagram shows the approximate temperature ranges for precipitation of secondary phases, where the most important are carbides, nitride, phases σ, χ, R, and γ_2_, and the influence of different alloying content on the kinetics of precipitation (Figure 1a,b). Higher alloying contents of Cr, Mo, W and Si cause extension of the precipitates’ range and shortening of the time dimension of TTP curves [7]. 

All major secondary phases are formed at temperatures above 500 °C. At temperatures above 500 °C, the chromium-rich ferrite, i.e., the α’ phase, dissolves into the γ phase. The onset of α’ phase decomposition at 500 °C limits the upper temperature during service. Below 500 °C, formation of precipitations is comparatively slow and of little importance for embrittlement [16]. 

An isothermal precipitation diagram for the 1.4462 DSS is shown in Figure 1b. Carbide and nitride precipitation begins at the relatively short time of 1–2 min at temperature. This is due to high solubility of carbon and nitrogen in the low nickel austenite phase as well as a retardation effect of nitrogen on carbide precipitation. Sigma and chi precipitation occur at higher temperatures [7,16]. 

### 2.3. Heat Treatment

One of the goals of the study was achieving the proper balance of ferrite and austenite phases to obtain suitable mechanical properties; therefore, three different treatments were carried out. Samples underwent the following heat treatments:

(a) raw material (reference sample);
(b) solution annealed at 900 °C for 1 h followed by slow cooling in the furnace;
(c) solution annealed at 1200 °C for 1 h followed by water quenching;
(d) solution annealed at 1200 °C for 1 h followed by water quenching then aging at 800 °C for 4 h followed by slow cooling in the furnace.

The choice of temperature range and method for the heat treatment of DSS was dictated by the prospect of introducing this material for the production of automatic weapons barrels. The study of structural changes under these conditions is aimed at understanding the processes taking place during the operation of the barrels, in particular the influence of the cooling rate on the austenite and ferrite phase fractions as well as the thermal properties of the material. The TTP diagrams show that slow cooling processes have the greatest influence on the kinetics of phase separation, especially the sigma phase.

Microstructure of the specimens was examined after chemical etching using Murakami, Picral and Oxalic reagents. ImageJ analysis was used to determine the amounts of various alloy phases. As a result, the percentage of ferrite and austenite in the samples was obtained as ratios of 75:25, 65:35 and 44:56, respectively. For the raw sample, it was not possible to determine the share of ferrite to austenite, because in the rolling process, oblong grains of small width, difficult to identify, were obtained.

### 2.4. Sample Preparation

Samples for testing thermal diffusivity in a form of cylinder with diameter *d* = 12.70 mm and thickness *l* = 1.99 mm were cut off from a piece of bar metal by a water-cooled cutting disc. In order to ensure high absorption of the pulse generated by the xenon flash lamp or laser flash, the sample surfaces were covered with a thin layer (2–3 μm) of graphite (GRAPHIT 33 Kontakt Chemie, Iffezheim, Germany). Density of 1.4462 DSS samples measured at room temperature using SARTORIUS analytical balance MSA125P-1CE-DA (readability [d]: 0.01 mg) was 7.77 g⋅cm^−3^. 

Samples for DSC investigations were cylinder-shaped with diameter *d* = 5.0 mm and were placed into a Al_2_O_3_ crucible, which was in turn inside a platinum crucible with a platinum lid (volume of Pt crucible: 85 µL). Sample masses of 1.4462 DSSs were 136.80 mg for 75:25 (α:γ), 77.41 mg for 65:35 (α:γ) and 127.86 mg for 44:56 (α:γ), respectively.

Samples for DIL testing had the shape of a 26 mm long cylinder with diameter of 5 mm cut from a bar by a water-cooled cutting disc. 

### 2.5. Surface Morphology Analysis and Vickers Micro-Hardness Measurements

Metallographic sample preparation involved cutting with an automatic precision cut-off machine and mounting in thermosetting bakelite resin, followed by grinding with 320 SiC paper, polishing with diamond pastes of 6, 3, 1 µm and a final polishing using 0.25 µm silica. The microstructures were analyzed using a digital (light) microscope, Keyence VHX-6000, as well as a scanning electron microscope Phenom Pro-X (Thermo Fisher Scientific, Waltham, MA, USA) with an acceleration voltage 15 kV equipped with an energy dispersive spectroscopy (EDS) chemical composition analyzer. Samples were examined after etching with Oxalic (C_2_H_2_O_4_), Picral (Picric acid and Ethanol) and Murakami’s reagent (100 mL water + 10 g NaOH + 10 g K_3_Fe(CN)_6_). Murakami’s reagent stains ferrite various shades of tan, depending on the length of immersion and the age of etching; carbides and intermetallics such as sigma stain black, and austenite remains white. A quantitative image analysis of the resulting light micrographs was performed using ImageJ (National Institute of Health). Distinction between the different phases was based on differences in grey values caused by the selective etching. No distinction could be made between un-etched phases such as σ and χ. The distinction between these phases was made by means of SEM, based on their contrast in Back-Scattered Electrons mode (BSE) (the χ-phase has a higher backscattering coefficient than σ as a result of its higher Mo content) and on their composition as determined by EDS. 

To validate the mechanical properties of 1.4266 DSS material, Vickers micro-hardness measurements were conducted with a load of 1000 g and loading time of 10 s for each single indentation using Qness Q10 A+ Micro Hardness Tester (ATM Qness GmbH, part of Verder Scientific, Maastricht, The Netherlands). The mean value was calculated from at least ten measurements for every sample.

### 2.6. Thermal Analysis

#### 2.6.1. LFA

The thermal diffusivity was determined using a HyperFlash LFA 467 light flash apparatus and LFA 427 laser flash apparatus (both from NETZSCH, Selb, Germany). The front surface of a plane-parallel sample was heated by a short energy pulse generated by a xenon lamp (LFA 467) or laser (LFA 427). From the resulting excess temperature on the rear face measured with an IR detector, the thermal diffusivity was calculated. In addition, for LFA 467, if a reference sample was used, the specific heat and thermal conductivity could also be calculated. The temperature range of the LFA 427 testing was between 25–1000 °C, and −50–480 °C for LFA 467. Both tests were carried out for the first heating. Argon at a flow rate of 50 mL⋅min^−1^ was used as an inert gas. At each temperature step during measurement of the thermal diffusivity, two shots were generated in order to average results. A standard Cape–Lehman model of heat transfer was used. This model takes into account the heat losses by radiation from the surfaces of the test sample. As a reference sample for the thermal diffusivity measurement, INCONEL 600 was used to enable determination of the specific heat and thermal conductivity of the tested 1.4462 DSS samples by a comparative method. The following relationship (1) was used to determine the specific heat capacity [17]:

(1) $$c_{p}^{s}\left(T\right)=\frac{T_{\infty }^{ref}}{T_{\infty }^{s}}\cdot \frac{\rho^{ref}}{\rho^{s}}\cdot \frac{d^{ref}}{d^{s}}\frac{Q^{s}}{Q^{ref}}\cdot \frac{V^{s}}{V^{ref}}\cdot \frac{d_{Orifice}^{2,s}}{d_{Orifice}^{2,ref}}c_{p}^{ref}\left(T\right)$$

where *d*: diameter, *V*: the signal amplitude gain, *T_∞_*: the corrected signal of the detector voltage taking into account heat loss, proportional to the adiabatic temperature increase, *ρ*: density, *Q*: pulse energy, *c_p_*: the specific heat under constant pressure; *s*: sample, *ref*: reference material; subscript *Orifice*: diameter of the IR detector measuring area. Taking into account Equation (1) and as calculated from LFA 467 thermal diffusivity values of the tested sample *a(T)*, the thermal conductivity $k^{s}\left(T\right)$ was calculated using Formula (2):

(2) $$k^{s}\left(T\right)=\frac{\rho_{0}}{{\left[1+\varepsilon \left(T\right)\right]}^{3}}\;\cdot \;a\left(T\right)\;\cdot \;c_{p}^{s}\left(T\right),$$

where *ε*(*T*) is the relative length change of the sample (thermal expansion).

#### 2.6.2. DIL

Thermal expansion measurements of 1.4462 DSSs were performed using a NETZSCH DIL 402 C pushrod dilatometer (NETZSCH, Selb, Germany) within the range of 200 °C to 1100 °C and a NETZSCH DIL 402 Expedis (NETZSCH, Selb, Germany) within the range of 50 °C to 500 °C. Nitrogen was used as an inert gas at a flow rate of 60 mL⋅min^−1^. The thermal expansion of the sample expressed by the coefficient of linear thermal expansion (CLTE) is in practice given in relation to the the initial length of the sample $L\left(T_{0}\right)$-CLTE*, which is also denoted by NETZSCH the physical alpha (α*), given by the Formula (3) [17]:

(3) $$CLTE^{*}\left(T\right)=\frac{1}{L\left(T_{0}\right)}\cdot \frac{dL\left(T\right)}{dT}=\frac{1}{L_{0}}\cdot \frac{dL\left(T\right)}{dT}$$

The heating/cooling rate (HR/CR) was 2 K⋅min^−1^.

#### 2.6.3. DSC

The thermal properties were determined using a differential scanning calorimeter, DSC 404 F1 Pegasus (NETZSCH, Selb, Germany). The temperature range of the DSC investigation was 25–1000 °C. Helium was used as an inert gas at a flow rate of 20 mL⋅min^−1^. Specific heat was calculated using the Cp ratio method based on the 3-DSC curves (baseline, sapphire line and tested sample line). In order to obtain a stable DSC signal, a double evacuation of the helium filling the furnace chamber and 15-min isothermal segments after each heating/cooling ones were applied. The heating/cooling rate (HR/CR) was 10 K⋅min^−1^.

## 3. Results and Discussion

### 3.1. Microstructural Analysis

#### 3.1.1. Raw Material

**Figure 2:**
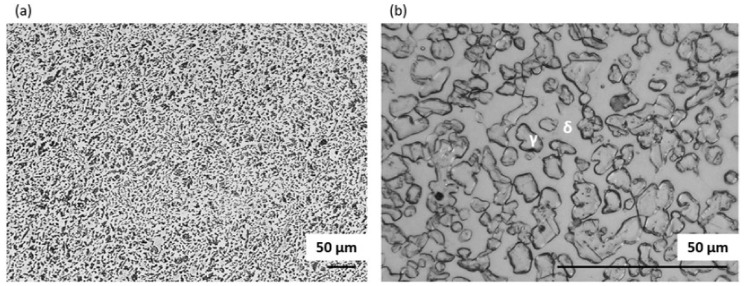
Optical image of microstructure of DSS 1.4462 in its as-delivered state: (**a**) etched with Murakami, (**b**) etched with Oxalic.

**Figure 3:**
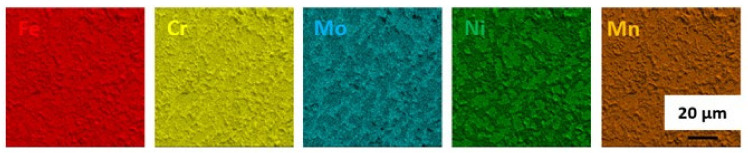
EDS maps of chemical element distribution in DSS 1.4462 in the as-delivered state.

A general view of the microstructure of the 1.4462 duplex steel in its as-delivered state is shown in Figure 2. The structure consisted of austenite (γ) islands embedded in a δ-ferrite matrix with no evidence of secondary precipitates. Figure 2 shows a quite isotropic microstructure as it is a transverse section; however, on the longitudinal section a banded texture of elongated grains could be observed as a consequence of deformation of material during the manufacturing process. The volume fraction of the γ phase as measured by quantitative metallography was approximately 0.55. The additive alloy elements were proportioned as can be seen in Figure 3. Increased levels of each element tend to be present in the phases they stabilize, so that austenite is enriched in nickel whereas ferrite is enriched in chromium and molybdenum.

#### 3.1.2. Solution Annealing at 900 °C for 1 h Followed by Slow Cooling in the Furnace

**Figure 4:**
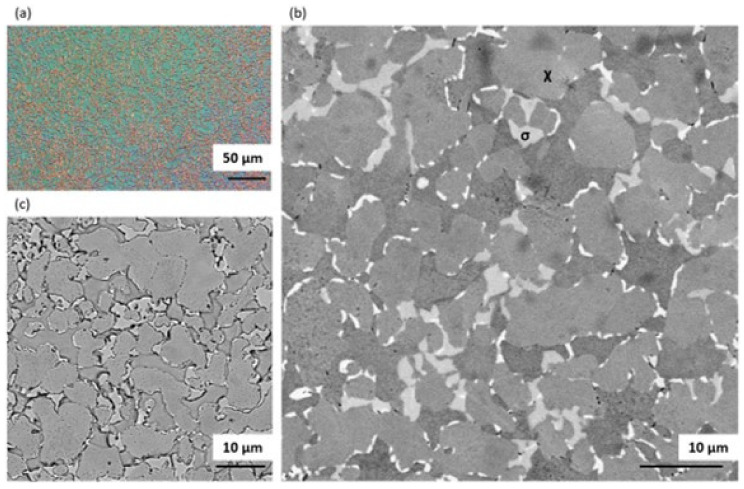
Images of the microstructure of a DSS 1.4462 solution annealed at 900 °C for 1 h followed by slow cooling in the furnace: (**a**) under a light microscope in polarized light, (**b**) SEM image—Picral etched, (**c**) SEM image—Murakami etched.

**Figure 5:**
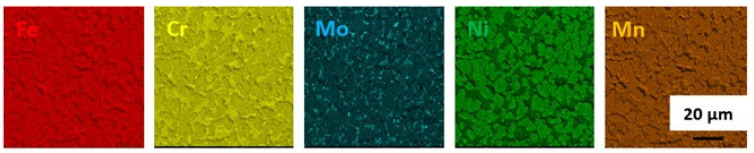
EDS maps of chemical element distribution in DSS 1.4462 solution annealed at 900 °C for 1 h followed by slow cooling in the furnace.

Heat treatment carried out at 900 °C for 1 h followed by slow cooling resulted in precipitation of both σ and χ phases (Figure 4). The obtained microstructure was composed of approximately 41% ferrite, 51% austenite, 6% sigma phase and 2% chi phase, and hence had the lowest volume fraction of ferrite (ferrite/austenite volume ratio δ/γ ≈ 0.79) among the tested heat treatments. An appreciable amount of intermetallic phase was present at the ferrite/austenite boundaries. It is worth noting that the formation of χ-phase is favoured at the beginning of annealing, as its crystal structure is similar to that of δ-phase. As annealing time increases, the χ-phase vanishes in favour of the σ-phase. EDS maps (Figure 5) clearly show the difference between χ- and σ-phase as χ-phase was much more enriched in Mo than σ-phase.

#### 3.1.3. Solution Annealing at 1200 °C for 1 h Followed by Water Quenching

**Figure 6:**
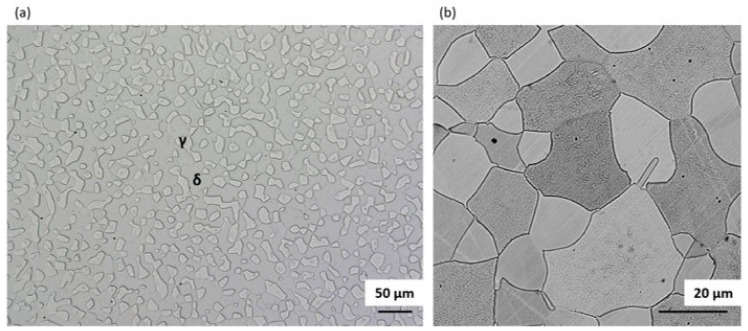
Images of the microstructure of a DSS 1.4462 solution annealed at 1200 °C for 1 h followed by water quenching: (**a**) optical image—Murakami etched, (**b**) SEM image—Murakami etched.

**Figure 7:**
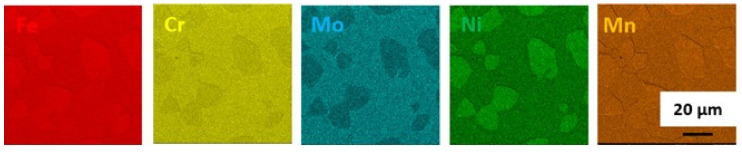
EDS maps of chemical element distribution in DSS 1.4462 solution annealed at 1200 °C for 1 h followed by water quenching.

The microstructure after solution heat treatment at 1200 °C for 1 h followed by water quenching is shown in Figure 6a,b. At this temperature, no intermetallic phases were noticed. Annealing at 1200 °C, however, resulted in strong grain growth and an increase in the δ volume fraction (δ/γ ≈ 3) as compared to the initial state. Moreover, an increase in grain size was observed compared to the material in its as-delivered state. The measured grain size of the γ-phase was approximately 20 µm and of the δ-phase was over 35 µm for the heat-treated sample, whereas in the initial state grain sizes were below 10 µm and below 15 µm, respectively. The map showing concentration of major alloying elements in ferrite and austenite analyzed using EDS is shown in Figure 7. It is clearly visible that Cr and Mo alloying elements enriched the ferrite phase while Ni was concentrated in the austenite phase. It is worth noting that since the volume fraction of ferrite phase was extremely high due to the annealing at a high temperature, the original ferrite forming elements, such as Cr and Mo, were spread over a larger volume and diluted, resulting in a decrease in Cr and Mo concentration in the ferrite phase. The EDS point analysis exhibited that Cr and Mo content in ferrite were approximately 21% and 4%, respectively.

#### 3.1.4. Solution Annealing at 1200 °C for 1 h Followed by Water Quenching Then Aging at 800 °C for 4 h Followed by Slow Cooling in the Furnace

**Figure 8:**
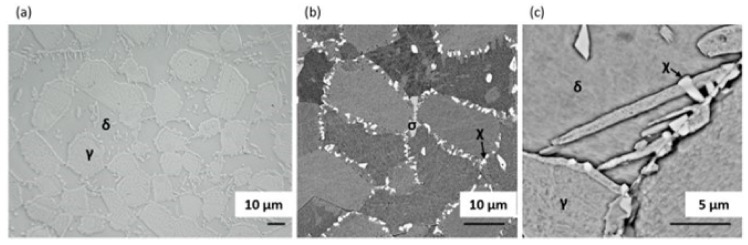
Images of the microstructure of a DSS 1.4462 solution annealed at 1200 °C for 1 h followed by water quenching and then aging at 800 °C followed by slow cooling in the furnace: (**a**) optical image—Murakami etched, (**b**) SEM image—Picral etched, (**c**) SEM image—Murakami etched.

**Figure 9:**
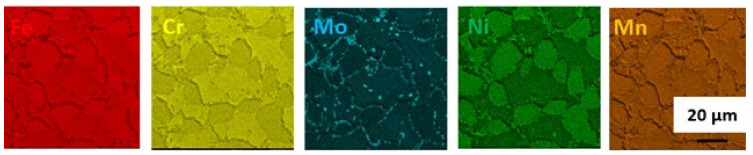
EDS maps of chemical element distribution in DSS 1.4462 solution annealed at 1200 °C for 1 h followed by water quenching then aging at 800 °C followed by slow cooling in the furnace.

The microstructure after solution annealing at 1200 °C and aging at 800 °C, consisting of approximately 62% ferrite, 34% austenite, 1% sigma phase and 3% chi phase, is shown in Figure 8. The ferrite/austenite ratio was δ/γ ≈ 1.86. Both σ and χ intermetallic phases can be easily distinguished in the SEM images, as the chi phase was richer than the sigma in heavy Mo and hence appears as the brightest phase. Furthermore, the differences among all phases are clearly visible on the EDS maps shown in Figure 9.

#### 3.1.5. Mechanical Properties

All measured hardness values of investigated samples are included in the Table 2. Hardness of the material in its as-delivered state was approximately 33 HRC (323 HV1). The highest hardness—38 HRC (367 HV1)—was observed for the sample that was annealed at 900 °C for 1 h followed by slow cooling in the furnace. This is in accordance with the literature and is caused by the occurrence of butterfly-shaped σ-phase in the microstructure [9]. Furthermore, this phenomenon is also associated with the amount of ferrite in the microstructure of DSS. Above 500 °C the amount of ferrite decreases slowly, while in the temperature range of 800–900 °C the decomposition of ferrite becomes extremely intensive, also correlating with the “nose point” of the C curve in the TTP diagram. The decrease in ferrite with the increase in austenite in the microstructure of the DSS leads to a rapid increase in hardness. Generally, body centered cubic (bcc) phases (ferrite) are much softer than face centered cubic (fcc) ones (austenite). Nevertheless, in this case fine sigma phase precipitations arise through bcc grains, strongly increasing hardness as a result of the Orowan dislocation hindering mechanism [18]. The heat treatments that were carried out for the next two samples—solution annealing at 1200 °C for 1 h followed by water quenching and solution annealing at 1200 °C for 1 h followed by water quenching, then aging at 800 °C followed by slow cooling in the furnace—resulted in a significant reduction in hardness compared to the material in its as-delivered state. As the heat treatment temperature rises, as in the case of solution annealing at 1200 °C, structural transformation leads to the consumption of σ-phase. As the degree of the recrystallization of the DSS microstructure increases, the constituent phases gradually become larger, resulting in decreasing hardness [19]. The slight increase in hardness observed for the solution annealed at 1200 °C and water quenched followed by aging at 800 °C with slow cooling, compared to the solution only annealed at 1200 °C, was induced by the precipitation of hard intermetallic phases.

#### 3.1.6. Discussion on Changes in the Microstructure

It is well known that the homogenization temperature and the cooling rate from this temperature affect the overall ferrite/austenite ratio, and are crucial in achieving the proper mechanical and corrosion properties of duplex steel [20,21,22,23,24]. Despite the fact that a great amount of work has been done to investigate the effect of cooling rate in the solid state of steels on the microstructure, phase distribution and formation of metastable phases [25], it is not fully understood how it affects the ferrite/austenite ratio in duplex steels. Generally, the ferrite percentage can be estimated using diagrams determined by Schaeffler [26], DeLong [27], Schoefer [28] and the Welding Research Council (WRC-1992) [29], which consider only the chemical composition, e.g., Cr and Ni equivalents. Therefore, these diagrams do not accurately predict the volume ratio of ferrite to austenite as they do not include cooling rates, which are crucial in microstructure formation. Along with ferrite and austenite there are three common intermetallic phases (the σ-, χ-, and Laves phases) that may be found in duplex steel. These phases reduce corrosion resistance as they are enriched in alloying elements such as Cr, Ni and Mo that are removed from matrix, and at the same time reduce the fracture toughness of the material due to precipitation at the grain boundaries. Thermodynamically, sigma phase is stable and forms on the Cr-rich site of the ternary phase diagram Fe–Cr–Ni at higher temperatures (900–1000 °C). Precipitation of sigma phase occurs between 650 and 1000 °C and complete precipitation takes several hours, leading to consumption of all the ferrite. The mechanism of its precipitation is a eutectoid reaction (δ → γ_2_ + σ), which starts at the δ/γ interface and grows into the ferrite as sigma phase becomes enriched in ferrite stabilizers (Cr, Mo and Si) [30]. It is also worth noting that the nucleation of sigma phase is heterogenous and does not depend on the crystallographic orientation relationships between ferrite and austenite. In contrast to ferrite, sigma phase is thermodynamically stable at elevated temperature [31]. When analysing the time-temperature-precipitation (TTP) diagram it can be perceived that the fastest precipitation rate for sigma phase is between 850 °C and 900 °C (Figure 8a) [11,32,33,34,35]. Furthermore, Phol et al. proved that the morphology of sigma phase is influenced by the precipitation temperature. At the temperature of 750 °C, a high density of single sigma nuclei forming a coral-like structure can be found [11]. The formation of this structure is related to low diffusion rates at this temperature, short diffusion distances and high local supersaturation. When the temperature reaches 950 °C, fewer sigma nuclei appear, while the diffusion rate increases, inducing formation of bigger and more compact precipitations. A similar relationship was found in our research; see Figure 5 and Figure 7.

Chi-phase, another common phase in duplex steel, is much more enriched in Mo and poorer in Cr compared to sigma-phase. Generally, the chi-phase precipitates near the ferrite/austenite interface but at times it can nucleate at ferrite/ferrite grain boundaries. It has been reported by Escriba et al. that chi-phase is stable at lower temperatures and can transform into sigma phase after aging for a long time [36]. Unlike sigma phase, chi phase is thermodynamically unstable. Moreover, at temperatures between 750 °C and 850 °C, precipitation of chi phase always occurs prior to that of sigma phase.

### 3.2. Thermal Properties Investigations

Thermophysical properties, i.e., specific heat, thermal diffusivity and thermal expansion, in the samples of 1.4462 DSS with the weight percentage ratios of ferrite to austenite of 75:25, 65:35 and 44:56, were tested in the temperature range of −50 °C–1000 °C. The first heating run allowed for the identification of the kinetics of precipitation processes, decomposition sequences and dissolution precipitations.

#### 3.2.1. LFA Investigations

**Figure 10:**
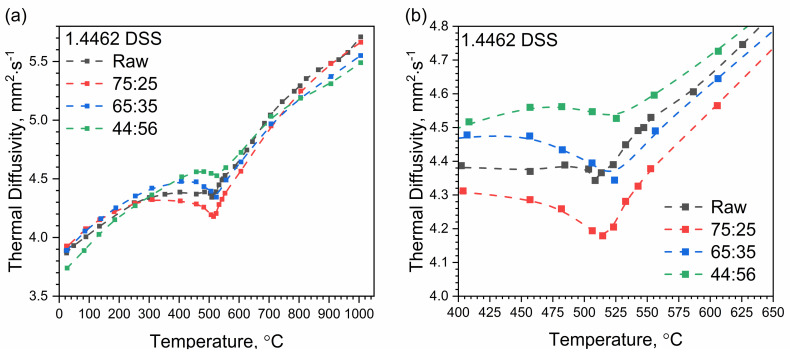
Thermal diffusivity as a function of temperature for 1.4462 DSSs with ferrite/austenite ratios of 75:25, 65:35, 44:56 and the raw sample, as obtained from the first heating runs on LFA 427: (**a**) in the range RT–1000 °C; (**b**) in the range 400–650 °C (segment of (**a**)).

Thermal diffusivity measurements were divided into two stages. First, measurements were made in the RT–1000 °C temperature range using the LFA 427 device. The temperature characteristic of thermal diffusivity was examined during the heating of the tested samples. Temperature characteristics of thermal diffusivity for 1.4462 DSS samples with the weight percentage ratios of ferrite and austenite of 75:25, 65:35, 44:56 and for the control sample are shown in Figure 10. At a temperature of approximately 500 °C, the chromium-rich ferrite, i.e., the α’ phase, dissolved, which resulted in a local minimum of thermal diffusivity for all samples. The effect was weak, being greatest for the sample with the 75:25 ferrite/austenite ratio. Additionally, as the ferrite content decreased, the local minimum moved towards a higher temperature (Table 3). In austenitic stainless steels, e.g., A310 steel, the thermal diffusivity characteristic is similar, i.e., thermal diffusivity increases in this temperature range from approximately 4 mm^2^/s to approximately 6 mm^2^/s, but this effect does not occur [17].

**Figure 11:**
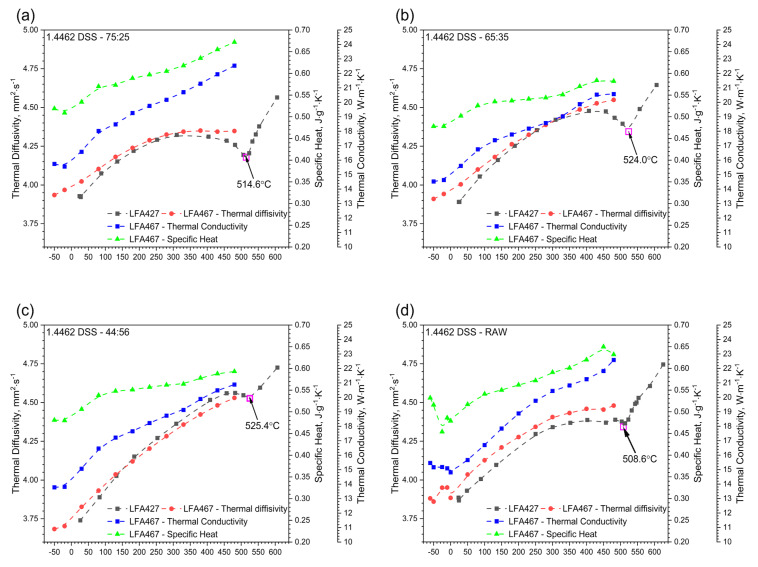
Thermal diffusivity, thermal conductivity and specific heat as a function of temperature for 1.4462 DSSs with ferrite/austenite ratios of 75:25, 65:35, 44:56 and the raw sample, obtained from the first heating runs on LFA 467: (**a**) 75:25; (**b**) 65:35; (**c**) 44:56 and (**d**) raw.

**Figure 12:**
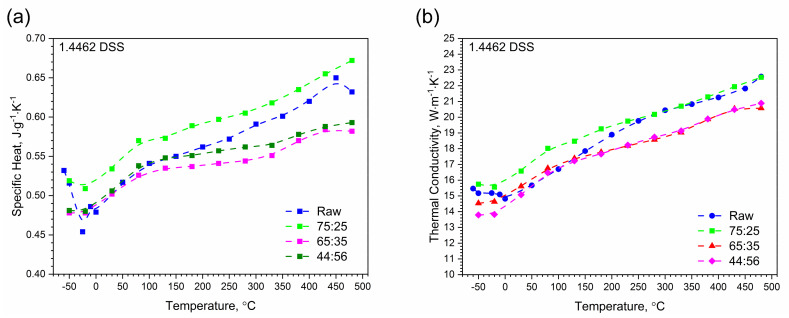
Comparison of specific heat and thermal conductivity of 1.4462 DSSs with ferrite/austenite ratios of 75:25, 65:35, 44:56 and the raw sample, obtained with the LFA 467 using the comparative method: (**a**) specific heat; (**b**) thermal conductivity.

**Figure 13:**
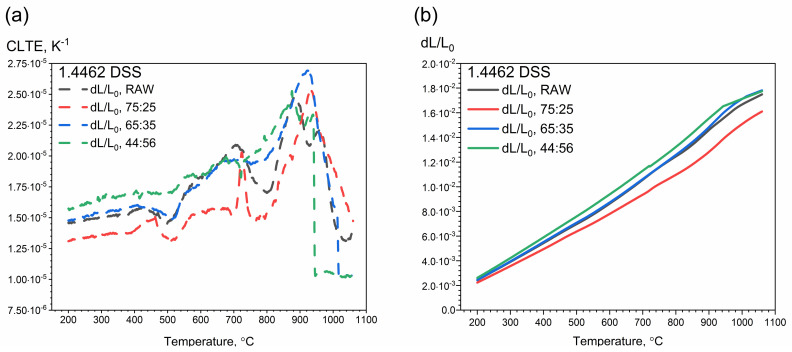
Thermal expansion and CLTE as a function of temperature for 1.4462 DSSs with ferrite/austenite ratios of 75:25, 65:35, 44:56 and the raw sample, obtained from the first heating runs on DIL 402 C: (**a**) CLTE(T); (**b**) dL/L_o_(T).

**Figure 14:**
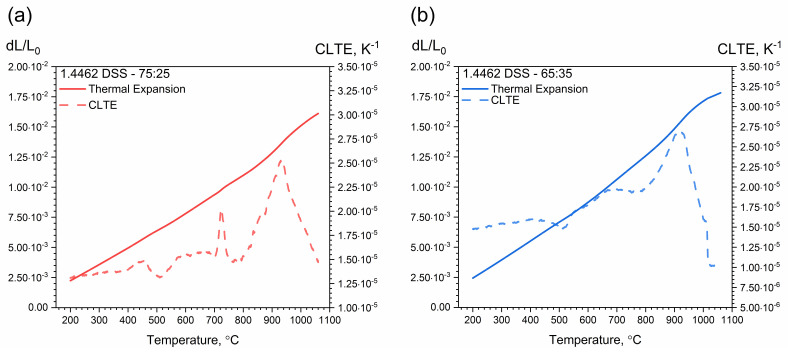

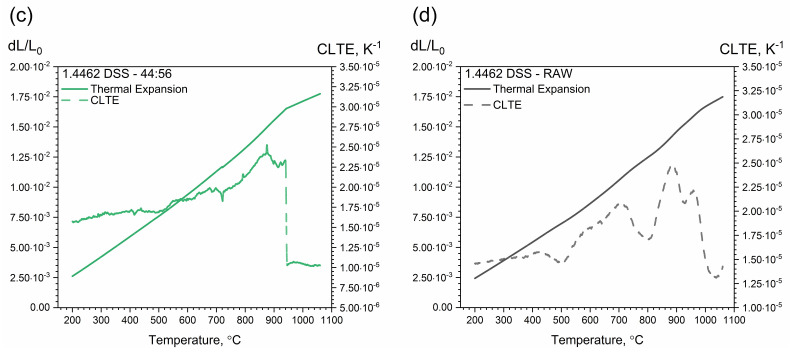
Thermal expansion and CLTE as a function of temperature for 1.4462 DSSs with ferrite/austenite ratios of: (**a**) 75:25, (**b**) 65:35, (**c**) 44:56 and (**d**) the raw sample, obtained from the first heating runs on DIL 402 C.

**Figure 15:**
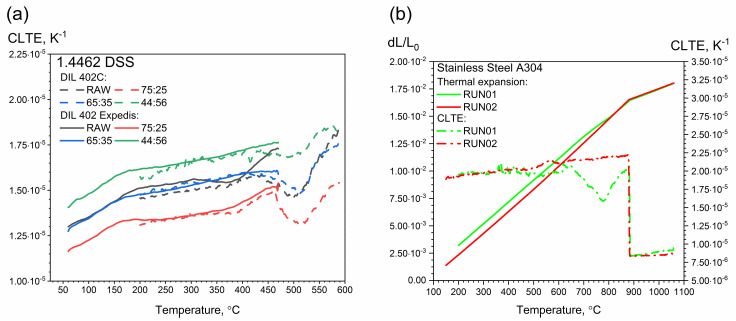
(**a**) Comparison of CLTE measurements as a function of temperature for 1.4462 DSS with ferrite/austenite ratios of 75:25, 65:35, 44:56 and the raw sample, obtained using DIL 402 Expedis and DIL 402 C. (**b**) Thermal expansion and CLTE as a function of temperature for A304, obtained using DIL 402 C [11].

In the second stage, thermal diffusivity was measured in the temperature range −50 °C–480 °C with the LFA 467 device. As before, thermal diffusivity tests were carried out while heating the measuring samples. The temperature characteristic of thermal diffusivity for samples of 1.4462 DSSs with ferrite/austenite ratios of 75:25, 65:35, 44:56 and for the raw sample, obtained with the use of LFA 467 and LFA 427, are shown in Figure 11, Figure 12, Figure 13, Figure 14 and Figure 15. The same figures also illustrate the relations of thermal conductivity and specific heat as functions of temperature.

There is good agreement between the thermal diffusivity characteristics obtained via LFA 427 and LFA 467. Figure 12b summarizes all thermal conductivity relationships as a function of temperature for samples of 1.4462 DSSs with ferrite/austenite ratios of 75:25, 65:35, 44:56 and for the raw sample obtained with the use of LFA 467, and Figure 12a shows all relationships of specific heat as a function of temperature. In the temperature range −50 °C–480 °C, the thermal conductivity of 1.4462 DSS samples increased linearly with temperature, reaching the highest level for ferrite/austenite equal to 75:25 and the raw sample, and the lowest level for ferrite/austenite equal to 65:35 and 44:56. The maximum relative discrepancies between them did not exceed 20% (Figure 12b). In the case of specific heat, the situation is similar, it means that the relative discrepancies are also approximately 20% (Figure 12a). The specific heat and thermal conductivity as a function of temperature for 1.4462 DSSs with ferrite/austenite ratios of 75:25, 65:35, 44:56 and for the raw sample were determined by the comparative method. The differences in thermal conductivity and specific heat in Figure 12a,b were due to the relatively low sensitivity of the LFA comparative method. The thermal conductivity, *k*, was calculated as a product of density, thermal diffusivity and specific heat to obtain data necessary for calculating heat transfer problems.

#### 3.2.2. DIL Investigations

Thermal expansion measurements were divided into two stages. First, measurements were made in the 200–1000 °C temperature range using the DIL 402 C device. The temperature characteristic of thermal expansion was examined during the heating of the tested samples. Temperature characteristics of thermal expansion for 1.4462 DSS samples with ferrite/austenite ratios of 75:25, 65:35, 44:56 and for the raw sample are shown in Figure 13. In the temperature range from 200 °C to approximately 450 °C, we observe a small linear increase in the CLTE values, but as the austenite content increased, the CLTE values became higher and higher (Figure 13). The highest value was for the ferrite/austenite ratio of 44:56. In austenitic stainless steels, e.g., in A304 steel, in this temperature range the CLTE values are approximately 2.00 × 10^−5^ K^−1^ [37].

At a temperature of approximately 500 °C, the chromium-rich ferrite, i.e., the α’ phase, dissolved, which resulted in a local minimum of thermal expansivity for all samples. This corresponds to the so-called “Brittleness of 475 °C”, which appears after annealing in the temperature range 400 °C–550 °C. It is hypothesized that this brittleness occurs as a result of dispersion strengthening with coherent precipitations of the rich chromium α’ phase. Such a process is possible because the Fe–Cr system has a range of insolubility below 600 °C in which the homogeneous solid solution can undergo spinodal transformation. Precipitations of the alpha prime phase create very fine spherical zones, which at higher temperatures take the shape of disks parallel to the ferrite planes. The phenomenon of dispersion hardening at the temperature of 475 °C increases with the content of chromium in steel [7,10,12,13,14,38,39,40]. At approximately 720 °C, there is a peak which corresponds to the calculated Curie temperature (706 °C) [13,14]. The peak is greatest with a ferrite content of 75%. At approximately 900 °C the σ phase dissolves with other carbides and nitrides [13]. This effect occurs with any ferrite content, but is strongest with a ferrite content of 75%, and weakest with a ferrite content of 44%. Above 900 °C, there is a rapid reduction in thermal expansion caused by the disappearance of austenite as a result of the transformation reaction of the austenitic phase γ_2_ into ferrite α. This is particularly visible in the sample with the highest content of austenite, i.e., 56% (Figure 14c).

As the ferrite content increased, the limit at which the rapid decrease in thermal expansion and CLTE was approached with ferrite content equal to:

44%—at the temperature of approximately 940 °C (Figure 14c);

65%—at the temperature of approximately 1020 °C (Figure 14b);

75%—at the temperature of approximately 1100 °C (Figure 14a);

raw—at the temperature of approximately 1000 °C (Figure 14d).

In the second stage, thermal expansion and CLTE measurements were made in the temperature range 50–500 °C with the DIL 402 Expedis device. As before, tests were carried out while heating the measuring samples. The test results were compared with the results obtained with the DIL 402 C (Figure 15a). No precipitation effects were observed in the temperature range 50–500 °C.

Additionally, Figure 15b illustrates thermal expansion and CLTE as a function of temperature for austenitic A304 stainless steel. The rapid decrease in thermal expansion and CLTE started as early as approximately 880 °C [37]. The CLTE values reached their maximum, i.e., approximately 2.25 × 10^−5^ K^−1^.

#### 3.2.3. DSC Investigations

**Figure 16:**
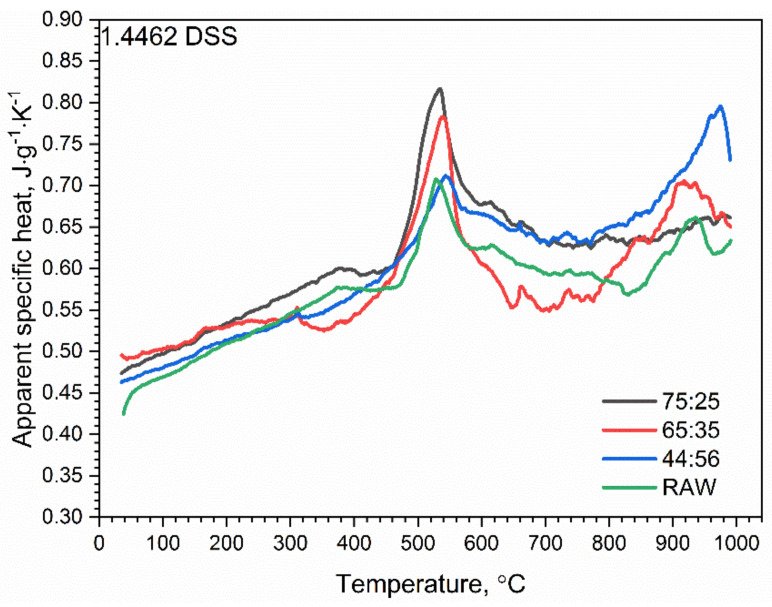
Temperature characteristics of apparent specific heat for 1.4462 DSSs with ferrite/austenite ratios of 75:25, 65:35, 44:56 and the raw sample.

**Figure 17:**
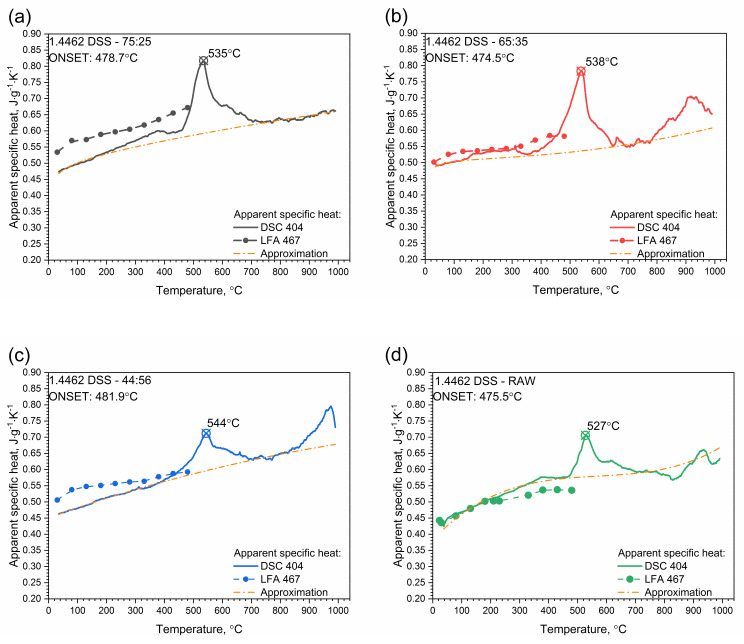
Temperature characteristics of apparent specific heat for 1.4462 DSS with ferrite/austenite ratios of: (**a**) 75:25, (**b**) 65:35, (**c**) 44:56 and (**d**) raw, obtained by the comparative method (LFA 467) and using DSC 404.

The results of specific heat investigations for 1.4462 DSS samples with ferrite/austenite ratios of 75:25, 65:35, 44:56 and for the raw sample are shown in Figure 16. At a temperature of approximately 500 °C, the chromium-rich ferrite, i.e., the α’ phase, dissolved and a first peak appeared for all 1.4462 DSS samples. A second peak appeared at approximately 900–950 °C, which was connected with the transformation reaction of the austenitic phase into ferrite one, but only for 1.4462 DSS samples with ferrite/austenite ratios of 65:35 and 44:56 and for the raw sample. For the 75:25 sample, this peak shifted towards higher temperatures, outside the measured range (Figure 17a).

For all measured 1.4462 DSS samples with ferrite/austenite ratios of 75:25, 65:35, 44:56 and for the raw sample, a correlation formula was proposed within the investigated temperature range (RT to 1000 °C). Figure 17 shows the fitting curves (dotted red lines) for the specific heat capacities of all of the 1.4462 DSS samples. The proposed formula has the following form (Equation (4)):

(4) $$c_{p}\left(T\left[℃\right]\right)=a_{0}+a_{1}T+a_{2}T^{2}+a_{3}T^{-\frac{1}{3}}\;\left[J\cdot g^{-1}\cdot K^{-1}\right],\;\;20{}^{\circ}C\leq T\leq 1000{}^{\circ}C$$

The values of coefficients *a_i_* are given in Table 4.

Knowledge of the thermophysical properties of barrel steels is necessary to carry out numerical simulations of heat transfer in the barrel of a rifle during shooting. In the calculation process, approximate specific heat characteristics are usually assumed, which are described by correlation formulas. In practice only the sensible heat capacity is assumed for the calculation of thermal conductivity so as not to duplicate the thermal effect associated with the phase transition, which can be accounted for via other thermophysical parameters such as thermal diffusivity or thermal expansion.

## 4. Conclusions

The heat treatment proposed by the authors and carried out experimentally led to the formation of three different microstructures in 1.4462 DSS: (a) a microstructure composed of approximately 41% ferrite, 51% austenite, 6% sigma phase and 2% chi phase with a ferrite/austenite volume ratio of δ/γ ≈ 0.79 for a DSS solution annealed at 900 °C for 1 h followed by slow cooling in a furnace, (b) a two-phase microstructure with a ferrite/austenite volume ratio of δ/γ ≈ 3 for a DSS solution annealed at 1200 °C for 1 h followed by water quenching, (c) a microstructure consisting of 62% ferrite, 34% austenite, 1% sigma phase and 3% chi phase with a ferrite/austenite ratio of δ/γ ≈ 1.86 for a DSS solution annealed at 1200 °C for 1 h followed by water quenching and then aging at 800 °C followed by slow cooling in the furnace.

For all samples the ferrite was enriched in Cr and Mo, while Ni was concentrated in the austenite. Further, chi phase was richer than sigma in heavy Mo.

Heat treatment greatly affected the hardness of the 1.4462 DSSs. As the solution treatment temperature rose, the mechanical properties of 1.4462 DSS varied according to a curve. For the 900 °C sample, the hardness was highest, reaching 33 HRC; for the 1200 °C sample hardness dropped to 25 HRC, while for the 1200 °C with additional aging at 800 °C sample, to 28 HRC. The differences in hardness were closely related to microstructural changes during heat treatment. Since the microstructure changed drastically after heat treatment, additional stress–strain tests were required. At this stage, it was difficult to comprehensively explain the change in mechanical responses using only hardness values. This problem was considered in the next research steps.

LFA research revealed slight differences in the thermometric characteristics of the thermal diffusivity at a temperature of approximately 500 °C for the 1.4462 DSS samples with ferrite/austenite ratios of 75:25, 65:35, 44:56 and for the raw sample. 

DIL tests confirmed the so-called “Brittleness of 475 °C”, which appeared after annealing the samples in the temperature range 400–550 °C, at a temperature of approximately 500 °C. In addition, the DIL examinations revealed further dissolution and precipitation processes that were not observed in the LFA and DSC thermograms, i.e., transformations at 720 °C, 900 °C and above 900 °C. 

DSC studies revealed only the dissolution of the *α^′^* phase at the temperature of approximately 500 °C and the transformation of γ_2_ into ferrite *α* and dissolution of the *σ* phase and other carbides and nitrides at approximately 900–950 °C. 

The results of the thermal property studies are summarized as follows:

(1) The analysis of LFA thermograms for the first heating using LFA 427 in the range RT–1000 °C allows us to conclude that:
  (a) near 500 °C, the chromium-rich ferrite, i.e., the *α^′^* phase, dissolved and a local minimum of thermal diffusivity for all samples appeared. This effect was greatest for the 75:25 ferrite/austenite sample. In the entire measuring temperature range, i.e., RT–1000 °C, LFA thermograms differed within 5%;
  (b) as the ferrite content decreased, the local minimum moved towards a higher temperature, most of all for the 44:56 ferrite/austenite sample (Table 2);
(2) The analysis of LFA thermograms from the first heating using LFA 467 in the range −50–480 °C shows that:
  (a) with respect to the specific heat determined from LFA 467 investigations for the first heating using the comparative method:
    an increase in the specific heat value was observed within the temperature range of −50–480 °C, and this was comparable for all samples, i.e., for ferrite/austenite ratios of 75:25, 65:35, 44:56 and for the raw sample.
  (b) with respect to the thermal conductivity from LFA 467 investigations for the first heating using the comparative method:
    in the temperature range of −50–480 °C, thermal conductivity increased linearly for all samples from 14 W⋅m^−1^⋅K^−1^ to approximately 22 W⋅m^−1^⋅K^−1^;
(3) The analysis of DIL thermograms using DIL 402 C for the first heating in the range 200–1000 °C shows that:
  (a) at a temperature of approximately 500 °C, the chromium-rich ferrite, or *α^′^* phase, dissolved. The CLTE change effect was greatest for the 75:25 ferrite/austenite sample and decreased with ferrite content;
  (b) at approximately 720 °C, there was a peak which corresponds to the calculated Curie temperature (706 °C). The peak was greatest with a ferrite content of 75%. The effect occurred with any ferrite content;
  (c) above 900 °C, there was a rapid reduction in thermal expansion. This was caused by the disappearance of austenite as a result of the transformation reaction of the austenitic phase into ferrite. It was particularly visible in the sample with the highest content of austenite, i.e., 56%, as it already appeared at 940 °C;
(4) DIL thermograms using DIL 402 Expedis for the first heating in the range 50–500 °C revealed:
  no precipitation effects were observed in the temperature range 50–480 °C.
(5) The analysis of DSC thermograms using DSC 404 F1 Pegasus for the first heating in the range of 25–1000 °C showed that:
  (a) at a temperature of approximately 500 °C, the chromium-rich ferrite, i.e., the *α^’^* phase, dissolved and a first peak in the apparent specific heat temperature characteristic appeared for all 1.4462 DSS samples;
  (b) at approximately 900–950 °C, for all but one sample, a second peak appeared, which was connected with the transformation of the austenitic phase into ferrite. For the 75:25 ferrite/austenite sample, this peak was shifted towards higher temperatures out of the measured range.

## Figures and Tables

**Table 1:** Chemical composition of 1.4462 DSS alloy [15].

Component	C	Mn	Cr	Ni	Mo	N	Cu	Si	Ti
Concentration [wt.%]	0.023	1.605	22.310	4.745	3.135	0.172	0.141	0.364	0.017
**Component**	**P**	**S**	**Fe**						
Concentration [wt.%]	0.025	0.001	Balance						

**Table 2:** Hardness of the investigated DSS.

	Initial	900 °C	1200 °C	1200 °C + 800 °C
HRC (HV1)	32.58 (323)	37.51 (367)	25.48 (269)	27.88 (285)

**Table 3:** Local minima of the thermal diffusivity as a function of temperature for 1.4462 DSSs with ferrite/austenite ratios of 75:25, 65:35 and 44:56.

Ferrite/Austenite	Raw	75:25	65:35	44:56
Minimum [°C]	508.6	514.6	524.0	525.4

**Table 4:** Coefficients for calculating specific heat capacity of 1.4462 DSS samples with ferrite/austenite ratios of 75:25, 65:35, 44:56 and for the raw sample based on Equation (4).

(a) Ferrite/austenite in the ratio 75:25			
Coefficient	Value	Coefficient	Value
$a_{0},\left[J\cdot g^{-1}\cdot K^{-1}\right]\;$	5.39 × 10^−1^	$a_{2},\left[J\cdot g^{-1}\cdot K^{-3}\right]\;$	−2.36 × 10^−8^
$a_{1},\left[J\cdot g^{-1}\cdot K^{-2}\right]\;$	1.71 × 10^−4^	$a_{3},\left[J\cdot g^{-1}\cdot K^{-\frac{4}{3}}\right]$	−2.54 × 10^−1^
(b) Ferrite/austenite in the ratio 65:35			
Coefficient	Value	Coefficient	Value
$a_{0},\left[J\cdot g^{-1}\cdot K^{-1}\right]\;$	5.51 × 10^−1^	$a_{2},\left[J\cdot g^{-1}\cdot K^{-3}\right]\;$	1.29 × 10^−7^
$a_{1},\left[J\cdot g^{-1}\cdot K^{-2}\right]\;$	4.95 × 10^−5^	$a_{3},\left[J\cdot g^{-1}\cdot K^{-\frac{4}{3}}\right]$	−2.01 × 10^−1^
(c) Ferrite/austenite in the ratio 44:56			
Coefficient	Value	Coefficient	Value
$a_{0},\left[J\cdot g^{-1}\cdot K^{-1}\right]\;$	4.56 × 10^−1^	$a_{2},\left[J\cdot g^{-1}\cdot K^{-3}\right]\;$	−8.23 × 10^−8^
$a_{1},\left[J\cdot g^{-1}\cdot K^{-2}\right]\;$	3.07 × 10^−4^	$a_{3},\left[J\cdot g^{-1}\cdot K^{-\frac{4}{3}}\right]$	−1.55 × 10^−2^
(d) Raw sample			
Coefficient	Value	Coefficient	Value
$a_{0},\left[J\cdot g^{-1}\cdot K^{-1}\right]\;$	5.39 × 10^−1^	$a_{2},\left[J\cdot g^{-1}\cdot K^{-3}\right]\;$	−1.74 × 10^−7^
$a_{1},\left[J\cdot g^{-1}\cdot K^{-2}\right]\;$	3.25 × 10^−4^	$a_{3},\left[J\cdot g^{-1}\cdot K^{-\frac{4}{3}}\right]$	−0.31 × 10^−9^

## Data Availability

The results of the research conducted as part of an internal university grant were not published as a report. This publication is the only place where our test results are presented.
